# Locally Injectable Chitosan/β-Glycerophosphate Hydrogel Doped with Triptolide–Human Serum Albumin Nanoparticles for Treating Rheumatoid Arthritis

**DOI:** 10.3390/ph17101312

**Published:** 2024-10-01

**Authors:** Pu Yao, Zirui Tan, Bangbi Weng, Xiaowen Wang, Hongping Wang, Ge Yang, Fengjun Sun, Ying Zhao

**Affiliations:** 1 School of Pharmacy& Bioengineering, Chongqing University of Technology, Chongqing 401320, China; upoay@stu.edu.cn (P.Y.);; 2 Department of Pharmacy, Southwest Hospital, Army Medical University (Third Military Medical University), Chongqing 400038, China

**Keywords:** rheumatoid arthritis, triptolide, thermosensitive hydrogel, intra-articular local treatment

## Abstract

Background: Rheumatoid arthritis (RA) tends to occur in symmetrical joints and is always accompanied by synovial hyperplasia and cartilage damage. Triptolide (TP), an extract from *Tripterygium*, has anti-inflammatory and immunomodulatory properties and could be used in the treatment of RA. However, its poor water solubility and the multi-system lesions caused by the use of this substance limit its clinical application. Therefore, it would be of great significance to assemble a composite nanoparticle hydrogel and apply it to a collagen-induced arthritis (CIA) mouse model to investigate the therapeutic effect and biosafety of this compound. Method: TP@HSA nanoparticles (TP@HSA NPs) were fabricated with a self-assembly method; a thermosensitive hydrogel loaded with the TP@HSA NPs (TP@HSA NP hydrogel) was prepared by using chitosan and beta- glycerophosphate (β-GP) and was then intra-articularly injected into CIA mice. The changes in joint swelling were measured with a digital caliper, and inflammation and cartilage damage were evaluated by using hematoxylin and eosin (H&E) and safranin O–fast green (SO&FG) staining, respectively. Results: TP@HSA NPs with an average diameter of 112 ± 2 nm were successfully assembled, and their encapsulation efficiency and drug loading efficiency were 47.6 ± 1.5% and 10.6 ± 3.3%, respectively. The TP@HSA NP hydrogel had a gelation temperature of 30.5 ± 0.2 °C, which allows for its injection at low temperatures and its sol–gel transformation under physiological conditions within 2 min, making it a suitable drug depot. The TP@HSA NP hydrogel was intra-articularly injected into CIA mice; it released TP locally and exerted anti-inflammatory and immunomodulatory effects, alleviating synovial inflammation and cartilage damage effectively. Conclusions: We successfully fabricated a TP@HSA NP-loaded thermosensitive hydrogel with good biosafety, which can release TP slowly for the treatment of RA. Our study provides a basis for the development of TP-based innovative preparations and has good application prospects.

## 1. Introduction

Rheumatoid arthritis (RA), an immune-related disease, often presents with the inflammation and swelling of symmetrical facet joints, resulting in synovial hyperplasia and cartilage damage. In addition to joint involvement, extra-articular manifestations involving all organ systems, including the nerves, the respiratory system, the liver, and the kidneys, also cause great distress to patients and significantly affect their quality of life [1,2]. Symptom relief and control over disease progression are still the focus of RA treatment, where non-steroidal anti-inflammatory drugs, glucocorticoids, and disease-modifying anti-rheumatic drugs are the mainstream therapeutic drugs at present [3,4]. In the meantime, the innovative application of traditional Chinese medicine (TCM) brings new hope for the prevention and treatment of RA.

Studies have shown that TP, which is extracted from TCM *Tripterygium*, has anti-inflammatory and immune-regulating properties [5,6]. A clinical study [7] showed that in RA patients, TP played an immunomodulatory role by inhibiting the production of IL2, IL4, and IFNγ in peripheral blood and the activation of CD4+ and CD8+ T cells. Another study [8] indicated that TP reduced the expression of CD80 and CD86 in IFNγ-induced dendritic cells (DC) and THP-1 cells, achieving immunomodulatory effects. In addition, TP was reported to inhibit the activation of microglia and the secretion of inflammatory factors in BV2 cells by upregulating miR96 [9]. It seems to be an ideal drug for RA treatment. However, its biological toxicity against the reproductive system, the liver, and the kidneys significantly narrows the treatment window and limits its wide application in clinical settings [10,11,12,13]. 

Innovative methods for TP detoxification represent a new direction to expand the scope of safe drug applications. With the development of artificial intelligence, smart drugs capable of responding to micro-physical and -chemical stimuli in the environment, such as changes in pH [14], temperature [15], nitric oxide [16], and reactive oxygen species (ROS) [17], have been extensively studied. Ren [18] et al. synthesized an acupoint nanocomposite hydrogel composed of TP and human serum albumin (HSA), through which TP was slowly released and delivered to the target site to relieve the symptoms of RA effectively. In another study, a hyaluronic acid hydrogel loaded with gold nanoparticles and low-dose TP was synthesized and intra-articularly injected under near-infrared irradiation to reduce TP toxicity and improve the specificity of treatment in RA [19]. An intratumoral administration thermosensitive hydrogel for sustained TP release showed lower systemic toxicity and better antitumor effects than free TP in 4T1 tumor-bearing mice [20]. It is noteworthy that auxiliary means such as acupuncture and infrared irradiation greatly reduce the compliance of patients [21,22].

HSA is commonly used as a drug carrier on account of its stability, non-toxicity, antigenicity, and biodegradability in vivo. However, it is difficult for hydrophobic drug molecules to enter its small internal hydrophobic chamber. Studies [23] have shown that the internal hydrophobic region of albumin can be expanded to contain more hydrophobic drug molecules by reducing its disulfide bonds with GSH, representing a further means for TP encapsulation. However, as the main lesions in RA concern the joints, systemic administration likely induces adverse reactions and has a significant negative impact on patients [24]. Local administration may be more suitable for the RA patient population. Research [25] has shown that chitosan (CS), a natural alkaline aminoglycan derived from n-acetylglucosamine and glucosamine residues by β-1, 4-glucoside linkage, presents high biocompatibility, low cytotoxicity, and biodegradability. It is worth noting that CS is not sensitive to temperature. It can be electrostatically adsorbed onto the amino group on beta-glycerophosphate (β-GP); however, when the electrostatic attraction is disrupted by the increase in temperature, chitosan begins to dehydrate, and the solution composed of CS and β-GP gradually changes into a gel [26]. A nanocomposite hydrogel drug delivery system used as a drug reservoir, with long-term enrichment and biodegradability at the target site, may be more conducive to RA treatment.

**Figure 1:**
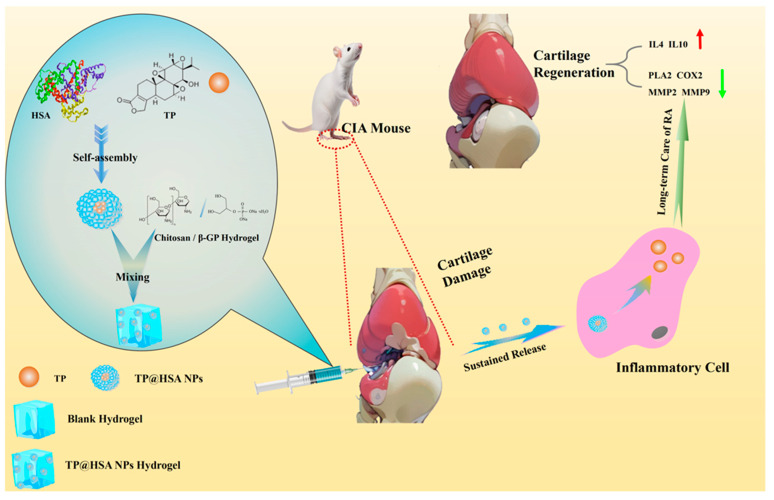
Hydrogel loaded with TP@HSA NPs was injected for RA treatment.

In this study, CS and β-GP were used to prepare thermosensitive hydrogel [27,28]. TP@HSA nanoparticles (TP@HSA NPs), which can increase the solubility of TP, were loaded on the hydrogel to form a nanocomposite hydrogel. The latter was intra-articularly injected into CIA mice to form a reservoir, improving the biosecurity of TP, which was slowly released to relieve inflammation and reduce cartilage damage (Figure 1). 

## 2. Results and Discussion

### 2.1. TP@HSA NP Assembly and TP@HSA NP Hydrogel Fabrication

**Figure 2:**
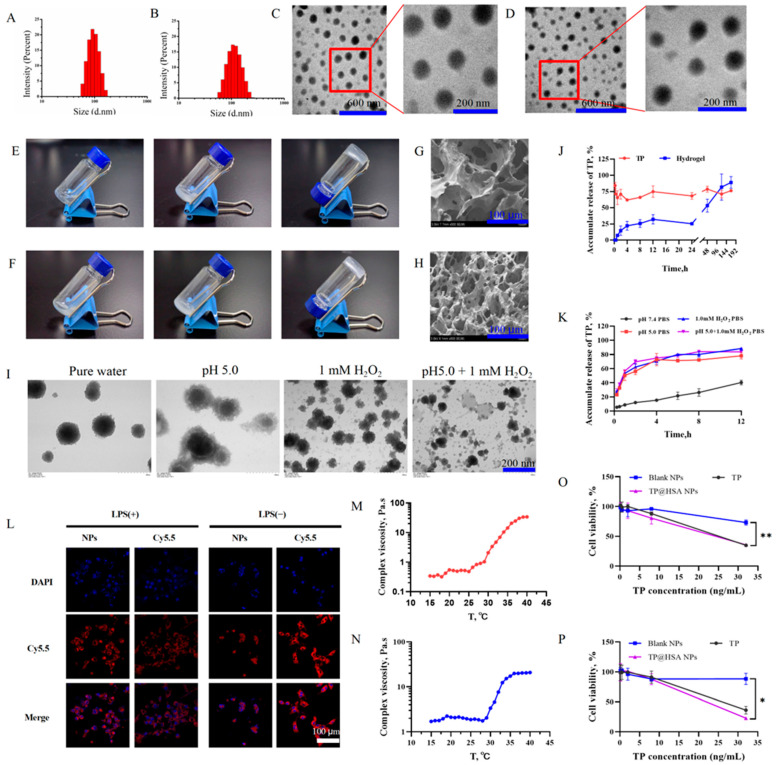
Characterization analyses of the TP@HSA NPs and TP@HSA NP hydrogel. The size distribution was determined with dynamic light scattering (DLS) (**A**) Blank NPs, and (**B**) TP@HSA NPs. The morphology of the (**C**) blank and (**D**) TP@HSA NPs was observed with TEM. The scale bars represent 600 nm in the original images and 200 nm in the enlarged sections. The vial tilting method was used to investigate the solution–gel transition of the (**E**) blank and (**F**) TP@HSA NP hydrogels. The morphology of the (**G**) blank and (**H**) TP@HSA NP hydrogels was observed with an SEM. The scale bar represents 100 µm. (**I**) The TEM-based analysis of the TP@HSA NPs morphology in acid and/or ROS medium. The scale bar represents 200 nm. The in vitro drug release behavior of TP from the (**J**) TP@HSA NP hydrogel in PBS buffer and the (**K**) TP@HSA NPs in various release media. (**L**) The uptake of TP@HSA NPs in LPS-activated and inactivated RAW264.7 cells. The gelation temperatures of the (**M**) blank and (**N**) TP@HSA NP hydrogels. The cell viability of RAW264.7 cells incubated at different concentrations of NPs for (**O**) 24 h and (**P**) 48 h, compared with Blank NPs, * *p* < 0.05; ** *p* < 0.01.

TP@HSA NPs were prepared by using a self-assembly/nanoprecipitation method [29,30]. The characterization results showed that the average diameters of the blank and TP@HSA NPs were 94 ± 1 nm (Figure 2A) and 112 ± 2 nm (Figure 2B), respectively; both types of NPs also had good solubility in water and had good dispersity with polymer dispersity index (PDI) between 0.05 and 0.15. As we can see from the results, the average diameter of the TP@HSA NPs was greater, indicating that the drug was successfully incorporated into the cavity of HSA. The morphology of the nanoparticles was observed by using transmission electron microscopy (TEM), and the results showed that both the blank nanoparticles (Figure 2C) and the TP@HSA NPs (Figure 2D) were uniformly dispersed and had a spherical shape. The morphology of the TP@HSA NPs in different media after 0.5 h of incubation was observed by using a TEM. The images revealed that the HSA in the outer layer of the NPs gradually collapsed/dispersed to release TP in acid and/or ROS medium (Figure 2I). The TP loading and encapsulation efficiency of the TP@HSA NPs were 10.6 ± 3.3% and 47.6 ± 1.5%, respectively (Table S1).

The vial tilting method [31] was used to investigate the colloidal properties of the hydrogels. The results reveal that the blank hydrogel (Figure 2E) and the TP@HSA NP-loaded hydrogel (TP@HSA NP hydrogel) (Figure 2F) transformed into a gel when subjected to an ice bath for more than 5 min but underwent sol–gel transformation at 25 °C in 2 min. Moreover, the results of the rheometrical study showed that the gelation temperatures of the blank and TP@HSA NP hydrogels were 29.8 ± 0.4 °C (Figure 2M) and 30.5 ± 0.2 °C (Figure 2N), respectively, indicating that the prepared hydrogels were temperature-responsive and could quickly form a “drug release depot” under physiological temperature conditions. The morphological characterization of the freeze-dried blank hydrogel (Figure 2G) and TP@HSA NP hydrogel (Figure 2H) performed with a scanning electron microscope (SEM) showed a three-dimensional network structure; as we can see from the figure, when the spherical NPs were loaded on the hydrogel, the resulting structure was tighter than that of the blank gel, indicating the successful preparation of the TP@HSA NP hydrogel.

### 2.2. TP Release from NPs and Hydrogel

Next, the in vitro drug release behavior of TP@HSA NPs from the TP@HSA NP hydrogel was investigated in PBS buffer (pH 7.4). As shown in Figure 2J, the TP@HSA NPs were gradually released, with up to 90% being released by the 7th day, indicating that nanoparticle release was slow and sustained and could achieve a longer dosing interval. The TP release behavior of the TP@HSA NPs was also evaluated under acidic and/or ROS conditions. As seen in Figure 2K, under acidic/ROS conditions, TP was completely released from the TP@HSA nanoparticles within 8 h, indicating that the structure of the nanoparticles was destroyed by the inflammatory microenvironment, allowing for the release of TP for RA treatment.

### 2.3. In Vitro Cytotoxicity Study of TP@HSA NPs

We evaluated the cellular biocompatibility of TP@HSA NPs with CCK kits, and the role of NPs in the inflammatory environment in vitro was investigated with RAW264.7 cells [32].

After incubation with TP or TP@HSA NPs for 24 h (Figure 2O) and 48 h (Figure 2P), clear cytotoxicity was observed at the concentration of 32 ng/mL, and statistically significant differences in cell viability were observed in the absence or presence of TP or TP@HSA NPs. When the concentration of TP was less than or equal to 8 ng/mL, the RAW264.7 cells showed similar cell viability to that after incubation with the blank NPs, demonstrating their suitable biocompatibility. 

### 2.4. Intracellular Uptake

Whether nanoparticles can be taken up by inflammatory cells is an important factor in determining their effectiveness in the body. We investigated the cellular uptake of Cy5.5 NHS-labeled NPs in lipopolysaccharide (LPS)-activated or inactivated RAW264.7 cells. As shown in Figure 2L, the fluorescence intensity of the Cy5.5 NHS-labeled NPs was stronger than that of free Cy5.5 NHS. Additionally, the internalized NPs in the LPS-activated RAW264.7 cells were present in a much higher volume than in the inactivated cells, indicating that the inflammatory environment induced by LPS could enhance the uptake of NPs in RAW264.7 cells. These results demonstrate that the inflammatory environment promoted the uptake of NPs into cells, thereby exerting anti-inflammatory effects.

### 2.5. In Vivo Degradation of Hydrogel

Biodegradability is an important criterion to evaluate whether a substance could be used as a drug carrier. Therefore, the degradation rate of the blank hydrogel in vivo was monitored [33]. A blank hydrogel solution prepared in an ice bath was subcutaneously injected into a mouse and transformed into gel at physiological temperature. The amount of residual hydrogel was weighed after separation from the skin at specific time points. As shown in Figure S1A, the residual hydrogel amount decreased with time until it was completely degraded on the 14th day; its morphology is presented in Figure S1B. The biodegradability and long-term retention properties indicate that the hydrogel we assembled is suitable for long-term depot drug carrier development. 

### 2.6. Biotoxicity

Many studies have shown that TP is highly biotoxic [10,34]; therefore, a systematic study was conducted to investigate the potential long-term toxicity of TP and the TP@HSA NP hydrogel in vivo. Healthy male Kunming (KM) mice were intra-articularly injected with TP or TP@HSA NP hydrogel at doses of 0.2, 0.5, and 1.0 mg/kg. All the mice in the 1.0 mg/kg TP group died within 3 days, while no signs of apparent weakness were observed in the other groups during 28 days of monitoring. 

The hematoxylin and eosin (H&E) staining of the major organs was used to investigate whether or not TP exposure caused tissue damage or lesions. As shown in Figure S2, compared with the normal group, the hepatocytes in the 1.0 mg/kg TP@HSA NP hydrogel and free TP (0.2 and 0.5 mg/kg) groups presented different degrees of cell hypertrophy. Moreover, the lymph node margin was blurred, and the white pulp structure in the spleen cells was destroyed. At the same time, the size of the renal cell tubule vacuoles was increased, and the lung cells presented varying degrees of inflammatory infiltration in the different groups. However, no apparent histopathological abnormalities or lesions were found in the blank and 0.2 mg/kg and 0.5 mg/kg TP@HSA NP hydrogel groups. All of these data suggest that no significant toxicity was induced by the hydrogels loaded with TP@HSA NPs at less than 0.5 mg/kg over 28 days.

### 2.7. Anti-Inflammation and Cartilage Degeneration Prevention Effects Following Injection of TP@HSA NP Hydrogel in CIA Mice 

The effects of anti-inflammation and inhibition of cartilage degeneration were evaluated by intra-articularly injecting the TP@HSA NP hydrogel. In addition to the TP@HSA NP hydrogel group, free TP, TP@HSA NPs, and blank hydrogel groups were set up for comparison. A volume of 100 μL of solution for each group was freshly prepared and injected at the injured site of the mice, while the normal mouse group was not subjected to any treatment.

**Figure 3:**
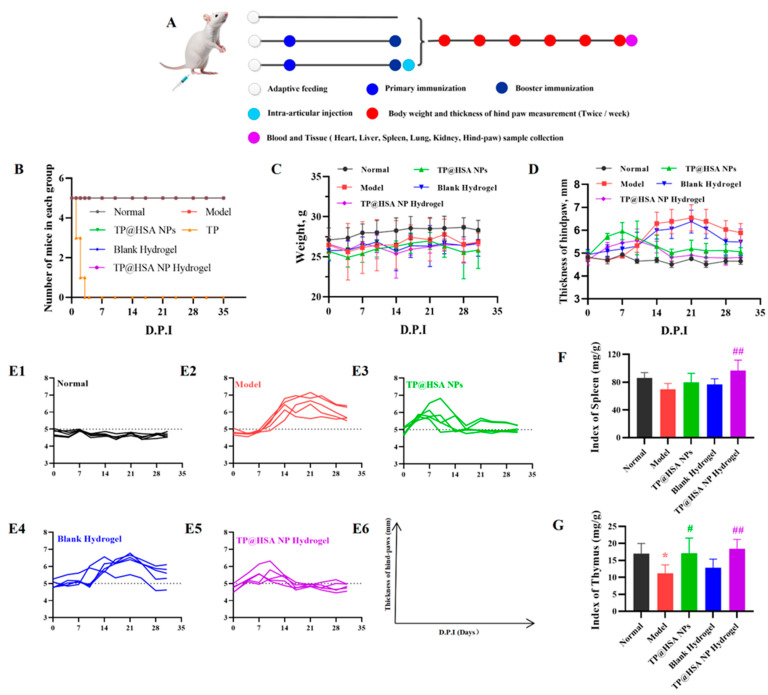
In vivo anti-arthritis efficacy evaluation of TP@HSA NP hydrogel in CIA mice. (**A**) Administration time after booster immunization. Mice received 0.5 mg/kg TP or isodose TP@HSA NPs or TP@HSA NP hydrogel formulations with intra-articular injection. (**B**) Survival curve, D.P.I stands for days post-immunization and (**C**) body weight changes in mice following different treatments (*n* = 5). (**D**) Hind-paw thickness changes in each group (*n* = 5). (**E**) Individual hind-paw thickness changes in mice under different treatments; (**E1**) Normal group, (**E2**) Model group, (**E3**) TP@HSA NPs group, (**E4**) Blank Hydrogel group, (**E5**) TP@HSA NP Hydrogel group, (**E6**) Horizontal and longitudinal annotations for (**E1**–**E5**). Immune organ indexes of (**F**) spleen and (**G**) thymus under different treatments (*n* = 5), compared with normal group, * *p* < 0.05; compared with Model group, # *p* < 0.05, ## *p* < 0.01.

The treatment of collagen-induced arthritis (CIA) mice lasted five weeks (Figure 3A). As shown in Figure 3B, the mice that were given 0.5 mg/kg free TP died within 3 days after booster immunization, and no deaths were recorded in the other groups. The body weight of all CIA mice underwent no noticeable changes during the therapy (Figure 3C). Since the index of arthritis is an important indication for clinical evaluation of RA, it was calculated, along with the thickness. Compared with the normal group, the arthritis indexes of the model and blank hydrogel treatment groups increased, while those of the mice that were treated with various TP-loaded preparations first increased within 3 days and subsequently decreased (Figure 3D), which may be ascribed to the bidirectional immunomodulatory effect of TP [5]. As shown in Figure 3E, the proportion of animals with a hind-paw thickness over 5 mm in the TP@HSA NP hydrogel group was the lowest among all groups and almost the same as the normal group, indicating that the TP@HSA NP hydrogel had the best therapeutic effect in the CIA mice. Additionally, the immune organ index of the TP@HSA NP hydrogel treatment group, including the spleen and thymus, was closer to that of the normal group (Figure 3F,G), indicating the favorable immunomodulatory effect of TP. 

**Figure 4:**
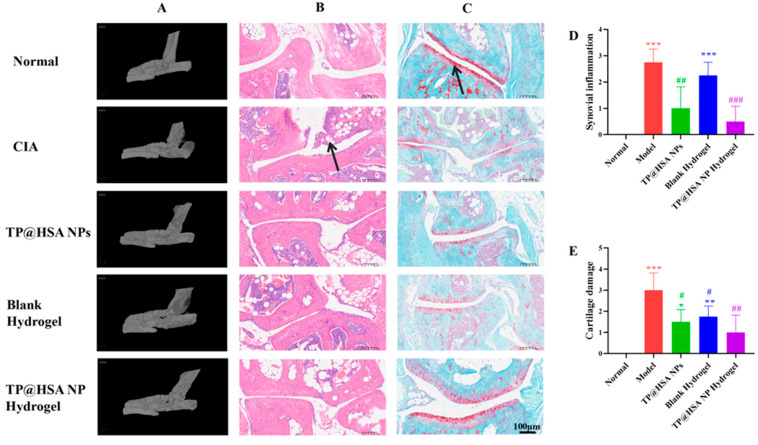
In vivo cartilage degeneration prevention efficacy evaluation of TP@HSA NP hydrogel in CIA mice. (**A**) Micro-CT of left hind paw. Histological sections with (**B**) H&E (4×) and (**C**) SO-FG (4×) staining of joints in different groups. Black arrow in (**B**): invasion of inflammation. Black arrow in (**C**): undamaged cartilage. Scale bar: 100 μm. Histopathologic scores assessed by two independent investigators of (**D**) synovial inflammation and (**E**) cartilage degradation. Compared with normal group, * *p* < 0.05, ** *p* < 0.01, *** *p* < 0.001; compared with Model group, # *p* < 0.05, ## *p* < 0.01, ### *p* < 0.001.

The mice were sacrificed after treatment, and the ankle joints were fixed for 24 h after dissection; then, micro-CT images were taken for morphological analyses (Figure 4A), and H&E (Figure 4B) and safranin O–fast green (SO&FG) (Figure 4C) staining were performed for pathological characteristic observation. Significant inflammatory infiltration and cartilage defects, including the disappearance of the characteristic red staining of glycosaminoglycans identified in healthy cartilage, were found in the model group, which showed considerably progressed CIA. However, the pathological status of the cartilage in each TP-loaded treatment group demonstrated well-maintained morphological characteristics. It is particularly noteworthy that the TP@HSA NP hydrogel group showed significant improvement in anti-CIA effects compared with the TP@HSA NPs group, as well as morphological similarity with normal cartilage; further, in this group, the cellular characteristics of chondrocytes were well-maintained, indicating that the inflammation and cartilage damage caused by CIA were better suppressed by the sustained release of the TP@HSA NP hydrogel, protecting against the possible cytotoxicity of TP, which was fast-released by the TP@HSA NPs. Simultaneously, the scores of inflammatory infiltration and cartilage injury assessed by two independent appraisers [32], also showed the same results (Figure 4D,E).

### 2.8. ELISAs

Studies [35,36] have shown that, in hypoxic and inflammatory environments, the degradation of the extracellular matrix could promote the secretion of matrix metalloproteinase (MMPs) and cytokines, which may result in cartilage degeneration. Additionally, studies have reported that TP can exert anti-inflammatory effects by selectively suppressing the expression of related factors in the arachidonic acid metabolic pathway [5,37]. Therefore, the alteration in pro- and anti-inflammatory cytokines in serum was evaluated in the final stage of the experiments to verify the regulatory effect of the TP@HSA NP hydrogel on inflammation in RA.

**Figure 5:**
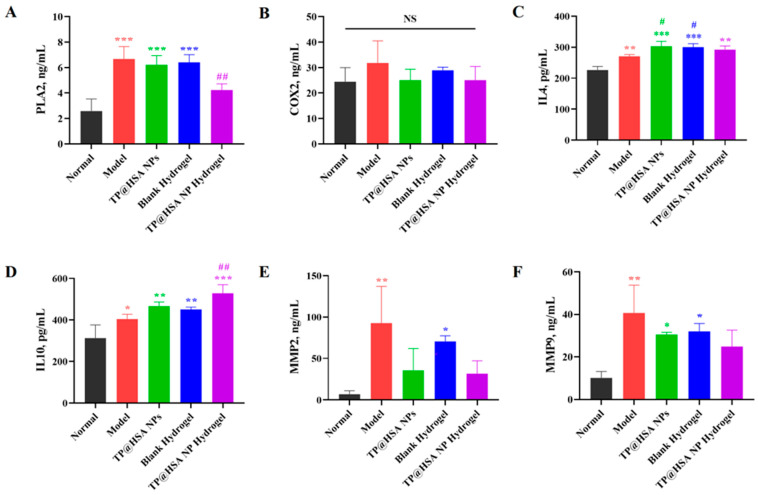
Serum concentrations of cytokines (*n* = 3). (**A**) PLA2; (**B**) COX2; (**C**) IL4; (**D**) IL10; (**E**) MMP2; and (**F**) MMP9. Compared with normal group, * *p* < 0.05, ** *p* < 0.01, *** *p* < 0.001; compared with Model group, # *p* < 0.05, ## *p* < 0.01.

As shown in Figure 5A, compared with the model group, the expression levels of phospholipase A2 (PLA2) in the arachidonic acid metabolic pathway were significantly decreased in the TP@HSA NP hydrogel group (*p* < 0.05), which could be related to the delayed entry into the bloodstream, and there were no significant differences in the expression of cyclooxygenase2 (COX2) (Figure 5B). The levels of anti-inflammatory cytokines interleukin 4 (IL4) (Figure 5C) and interleukin 10 (IL10) (Figure 5D) increased in the TP@HSA NP hydrogel group, and noticeably decreased levels of pro-inflammatory cytokines MMP2 (Figure 5E) and MMP9 (Figure 5F) were observed. Studies [35,38] have shown that the lowering of MMP and cytokine levels may demonstrate the successful prevention of cartilage degeneration. The results indicate that the TP@HSA NP hydrogel exerted a good anti-inflammatory effect on RA.

## 3. Materials and Methods

### 3.1. Materials

#### 3.1.1. Reagents and Instruments

Triptolide (TP) was supplied by Chengdu Herbpurity Co., Ltd. (L-004, Chengdu, Sichuan, China). Human serum albumin (HSA), L-Glutathione reduced (GSH; G8180), and streptomycin–penicillin liquid (P1400) were obtained from Solarbio life science (A8230, Beijing, China). Chitosan (CS; degree of deacetylation ≥ 95% and viscosity of 100–200 mpa.s; C804726) and β-glycerophosphate (β-GP; D859328) were obtained from Shanghai Macklin Biochemical Technology Co., Ltd. (Shanghai, China). Fetal bovine serum and Dulbecco’s modified Eagle’s medium (DMEM) were purchased from HyClone Inc. (Waltham, MA, USA). 4′,6-Diamidino-2-phenylindole (DAPI), and Enhanced Cell Counting Kit-8 were supplied by Beyotime Biotechnology Co., Ltd. (Shanghai, China). Cyanine5.5 NHS ester was purchased from Duofluor Inc. (D10013, Wuhan, Hubei, China). Complete Freund’s adjuvant (7001), incomplete Freund’s adjuvant (7002), and immunization-grade bovine type II collagen solution (20,022) were obtained from Chondrex, Inc. (Woodinville, WA, USA). 

H&E and SO&FG staining kits were obtained from Servicebio (Wuhan, China). IL4 (LCSED20186), IL10 (LCSSC20162), MMP2 (LCSED20378), MMP9 (LCSED20381), PLA2 (LCSED28124), and COX2 (LCSED23084) EILSA kits were purchased from LunChangShuo Biotech (Xiamen, China).

In this study, we used the following instruments: a Sorvall ST16R refrigerated centrifuge (ThermoFisher Scientific, Waltham, MA, USA), Secural25-1CN/SQP electronic scales (Beijing Sartorius Instrument εt System Engineering Co., Limited, Beijing, China), a Synergy H1 multifunctional enzyme marking instrument (Gene company limited, Shanghai, China), a 2690 high-performance liquid chromatography (HPLC; Waters Corporation, Milford, MA, USA), an LSM780 laser scanning confocal microscope (Carl Zeiss, Oberkochen, Germany), a ZetasizerNanoZSP nanoparticle size analyzer (Malvern Panalytical Ltd., Malvern, UK), a FEI Tecnai G2 12 transmission electron microscope (TEM; ThermoFisher Scientific, Waltham, MA, USA), a CLARA scanning electron microscope (SEM; TESCAN, Brno, Czech Republic), a T10 basic homogenizer (IKA, Staufen, Germany) and a SKYSCAN1272 for in vitro conical microfocal computerized tomography (micro-CT; Bruker, Kontich, Belgium).

#### 3.1.2. Cells

Mouse mononuclear macrophage leukemia RAW264.7 cells were obtained from Servicebio (Wuhan, China). The cells were incubated in high-glucose DMEM +10% fetal bovine serum (FBS) +1% penicillin–streptomycin (P/S) in a 37 °C constant-temperature incubator containing 5% CO_2_.

#### 3.1.3. Animals

KM mice or Balb/c mice (male; six to eight weeks old; ~20 g) were supplied by the experimental animal center of Army Medical University (Chongqing, China; License No. SYXK (Chongqing) 2022-0018). The animal experiment was approved by the animal ethics committee of Chongqing University of Technology (2023; Review No. 35). The animals were housed in a specific pathogen-free (SPF-level) room, and the feeding and experimental operation processes were in accordance with the regulations for the administration of affairs concerning experimental animals.

### 3.2. TP@HSA NP Assembly 

According to previous studies [18,23], 15 mg of HSA and 7.5 mg of GSH were added to 1 mL of water and mixed at 37 °C for 1 h. Then, 4 mg of TP dissolved in 2 mL of ethanol was added to the mixed solution, which was subjected to rapid agitation for 3 min; the self-assembly of TP@HSA NPs was performed at a low speed for 1 h. Finally, the organic solvent was removed by using a rotary evaporator at 37 °C (vacuum pressure, 0.08 MPa), and the nanoparticles were obtained with 10 kDa ultrafiltration. The blank NPs without TP were obtained in the same way.

A volume of 10 μL of nanoparticles was dispersed in 1 mL of water (pH 7.0) to determine the size and PDI with ZetasizerNanoZSP at an angle of 90°. The morphology of the nanoparticles was observed with a TEM (accelerating voltage of 100 kV and resolution of 0.38 nm). The encapsulation efficiency (EE) and drug loading (DL) of the nanoparticles were determined with high-performance liquid chromatography (HPLC). Briefly, 20 μL of NPs were added to 980 μL of methanol and centrifuged at 15,000 rpm for 5 min, and the supernatant sample was detected with 1.0 mL·min^−1^ acetonitrile/water (50/50) and an absorption wavelength of 218 nm.

EE (%) = Amount of drug in nanoparticles (mg)/Dosage (mg) × 100%

DL (%) = Amount of drug in nanoparticles (mg)/Weight of nanoparticles (mg) × 100%

To investigate the stability of the TP@HSA NPs under pH/H_2_O_2_ conditions, the morphological changes in the TP@HSA NPs were observed with a TEM after incubation with pure H_2_O, at pH 5.0, with 1 mM H_2_O_2_, and at pH 5.0 with 1 mM H_2_O_2_ for 0.5 h.

### 3.3. TP@HSA NP Hydrogel Fabrication

A total of 100 mg of chitosan was dissolved by adding 5 mL of 0.1 M acetic acid solution and stirring overnight in the dark. Under ice-bath conditions, a β-GP solution with a mass fraction of 60% and the TP@HSA NPs were added to a 2% CS solution, which was continuously stirred for 30 min to obtain a hydrogel loaded with TP@HSA NPs [31]. The blank hydrogel without TP@HSA NPs was operated in parallel. The sol–gel transition was observed by performing vial tilting; then, the gelation temperature of the blank and TP@HSA NP hydrogels was monitored with a rheometer. Finally, their morphology was observed with an SEM.

### 3.4. TP Release from TP@HSA NPs and TP@HSA NP Hydrogel

The TP@HSA NPs were added to a dialysis tube (3500 Da) and placed into 40 mL of different PBS buffers (pH 7.4, pH 5.0, 1.0 mM H_2_O_2_, and pH 5.0/1.0 mM H_2_O_2_) containing 1.0% Tween. The TP@HSA NP hydrogel and free TP solution were added to a dialysis tube (3500 Da) and placed into 40 mL of PBS buffer (pH 7.4) containing 1.0% Tween.

Under constant shaking at 37 °C, 4 mL of the release buffer was taken out, and an equivalent volume of fresh buffer was added at different time points (0.25, 0.5, 1, 2, 4, 8, and 12 h for the TP@HSA NPs and 0.25, 0.5, 1, 2, 4, 8, 12, 24, 48, 120, and 168 h for the TP@HSA NP hydrogel). Then, equal amounts of the above-release liquid and methanol were mixed, and the supernatant was detected by using the aforementioned HPLC method.

### 3.5. Cell Viability

The in vitro cytotoxicity of the TP@HSA NPs against mouse mononuclear macrophage leukemia cells (RAW264.7) was evaluated with a CCK-8 assay. Briefly, LPS (1 μg·mL^−1^)-activated for 24 h or inactivated RAW264.7 cells (1 × 10^4^) that adhered to a 96-well plate were treated with different concentrations of the drug (0.125, 0.5, 2.0, 8.0, and 32.0 ng·mL^−1^) and cultured in an incubator for 24 h and 48 h. Then, the cells were incubated with CCK8 solution for 30 min. Finally, the OD_450_ value was determined by using a multifunctional enzyme labeler.

### 3.6. Intracellular Uptake

LPS (1 μg·mL^−1^)-activated for 24 h or inactivated RAW264.7 cells (1 × 10^5^) that adhered to a confocal culture dish were incubated with different types of substances (Cy5.5 and Cy5.5-labeled NPs) [39] in the dark for 4 h. Subsequently, the cells were fixed in 4% paraformaldehyde for 15 min and washed with PBS three times. Then, DAPI was added for staining for 5 min and washed out with PBS. Finally, the uptake of the NPs by the RAW264.7 cells was observed with a confocal microscope.

### 3.7. Degradation of Hydrogel

A volume of 0.1 mL of blank hydrogel was subcutaneously injected into the back of the mice; the hydrogel was removed after 0, 1, 2, 4, 7, 10, and 14 days and weighed and photographed to observe its degradation in vivo.

### 3.8. Long-Term Toxicity

The KM mice were administered 0.2 mg/kg TP, 0.5 mg/kg TP, 1.0 mg/kg TP, blank hydrogel, 0.2 mg/kg TP@HSA NP hydrogel, 0.5 mg/kg TP@HSA NP hydrogel and 1.0 mg/kg TP@HSA NP hydrogel (0.1 mL) in the joint cavity. At the same time, the normal group did not receive any treatment. On the 28th day, the mice were sacrificed in the 0.3% carbon monoxide, and heart, liver, spleen, lung, and kidney tissues were collected for H&E staining.

### 3.9. In Vivo

CIA was induced in 6–8 w Balb/c male mice according to the collagen-induced mouse arthritis experimental protocol provided by Chrondrex. Briefly, equal amounts of complete adjuvant (CFA) and immunization-grade bovine type II collagen solutions were blended in a tube to form an emulsion with a high-speed agitator in an ice bath. Then, 0.1 mL of emulsion containing 100 μg of collagen was subcutaneously injected into the mice to achieve primary immunization. On the 21st day, a booster immunity emulsion containing incomplete adjuvant (IFA) and collagen solution was prepared with the same method and subcutaneously injected at a dose of 0.1 mL to complete booster immunization.

#### 3.9.1. Pharmacodynamics

Mice in the normal and CIA groups did not receive treatment. The TP, TP@HSA NPs, blank hydrogel, and TP@HSA NP hydrogel groups were treated with intra-articular injection at the ankle with medicine containing 0.5 mg/kg TP. The weight, arthritis score, and hind-paw thickness of the mice were recorded twice a week.

#### 3.9.2. Micro-CT

The mice were sacrificed on the 35th day. Their hind paws were dissected and fixed in 4% paraformaldehyde for at least 24 h. Micro-CT scanning was used to observe the degradation of the bone. The scanning parameters were set as follows: the voltage was 85 KV, the scanning resolution was 10.141270 μm, and the exposure time was 384 ms. Finally, the selected area was 3D-reconstructed with CTvox.

#### 3.9.3. Bone Tissue Staining

After having been fixed in 4% paraformaldehyde for 24 h, the hind paws were immersed in 10% EDTA solution for 21 days to achieve decalcification. Then, H&E staining and SO&FG staining were used to observe synovial infiltration and cartilage damage, respectively [32].

#### 3.9.4. Serum ELISAs

According to the experimental protocols provided by the manufacturer, the IL4, IL10, MMP2, MMP9, PLA2, and COX2 concentrations in mouse serum were quantitatively determined.

### 3.10. Statistical Analysis

All data are reported as the means ± standard deviations (SDs) of independent experiments. The statistical analyses were performed with the unpaired two-tailed Student’s *t*-test for two groups and one-way analysis of variance (ANOVA) with Tukey’s multiple comparisons test for multiple-group comparisons. Statistical significance was defined as follows: compared with the normal group, * *p* < 0.05, ** *p* < 0.01, *** *p* < 0.001 and **** *p* < 0.0001; compared with the model group, ^#^ *p* < 0.05, ^##^ *p* < 0.01, ^###^ *p* < 0.001 and ^####^ *p* < 0.0001.

## 4. Conclusions

Self-assembled TP@HSA NPs were loaded on temperature-sensitive hydrogel consisting of CS and β-GP. The TP@HSA NP hydrogel remains in a solution state at a low temperature, which makes it convenient to inject, and quickly undergoes the sol–gel transformation under physiological conditions, forming a depot that can release TP slowly and increase the retention time. The application of TP@HSA NP hydrogel diminished joint swelling in CIA mice and reduced osteophytes by slowly releasing TP effectively. Not only local pathological features were improved, but also inflammatory factor expression was balanced, and the toxic and side effects of TP were significantly reduced. In summary, the TP@HSA NP hydrogel that we assembled demonstrated enhanced efficacy and improved safety, providing a new means for the application of TP.

## Data Availability

Data are contained within the article and Supplementary Materials.

## Supplementary Materials

The following supporting information can be downloaded at: https://www.mdpi.com/article/10.3390/ph17101312/s1, Table S1. Physicochemical properties of various nanoformulations. Figure S1. Biodegradation study of blank hydrogel. (A) Time-dependent hydrogel degradation after subcutaneous injection in mouse. (B) Visualization of hydrogel changes. Figure S2. Hematoxylin and eosin (H&E) staining for biotoxicity of TP and TP@HSA NP hydrogels in each group. Black arrow: cell hypertrophy. Green arrow: blurred lymph node margin. Blue arrow: invasion of inflammation. Red arrow: size increased renal cell tubule vacuoles. (4×; scale bar: 200 μm).
