# Supplementary material for: Locally Injectable Chitosan/β-Glycerophosphate Hydrogel Doped with Triptolide–Human Serum Albumin Nanoparticles for Treating Rheumatoid Arthritis
Source: Pharmaceuticals (Basel). 2024 Oct 1;17(10):1312. doi: 10.3390/ph17101312 (PMC11510276; doi:10.3390/ph17101312)
Supplement: Supplementary file 1 [file pharmaceuticals-17-01312-s001.zip › pharmaceuticals-3173741-supplementary.pdf]

Table S1. Physicochemical properties of various nanoformulations.

Nanoformulation	Size (nm)	Drug Loading (w/w %)	Encapsulation Efficiency (%)
Blank NPs	94 ± 1	-	-
TP@HSA NPs	112 ± 2	10.6 ± 3.3%	47.6 ± 1.5%

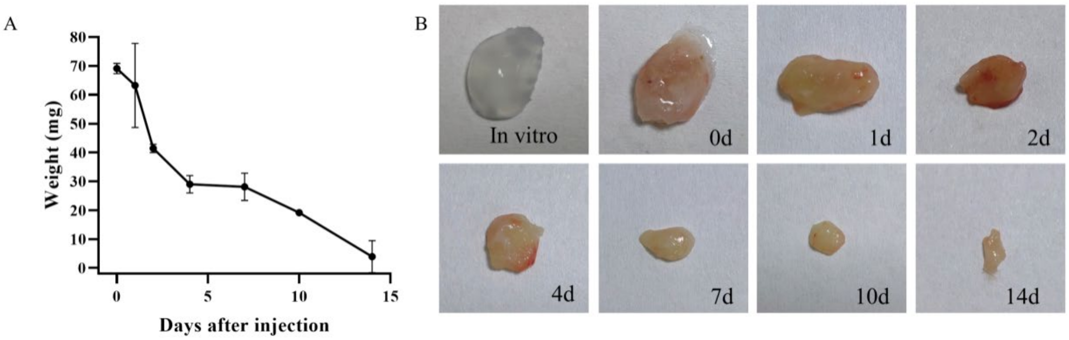

Figure S1. Biodegradation study of blank hydrogel. (A) Time-dependent hydrogel degradation after subcutaneous injection in mouse. (B) Visualization of hydrogel changes.

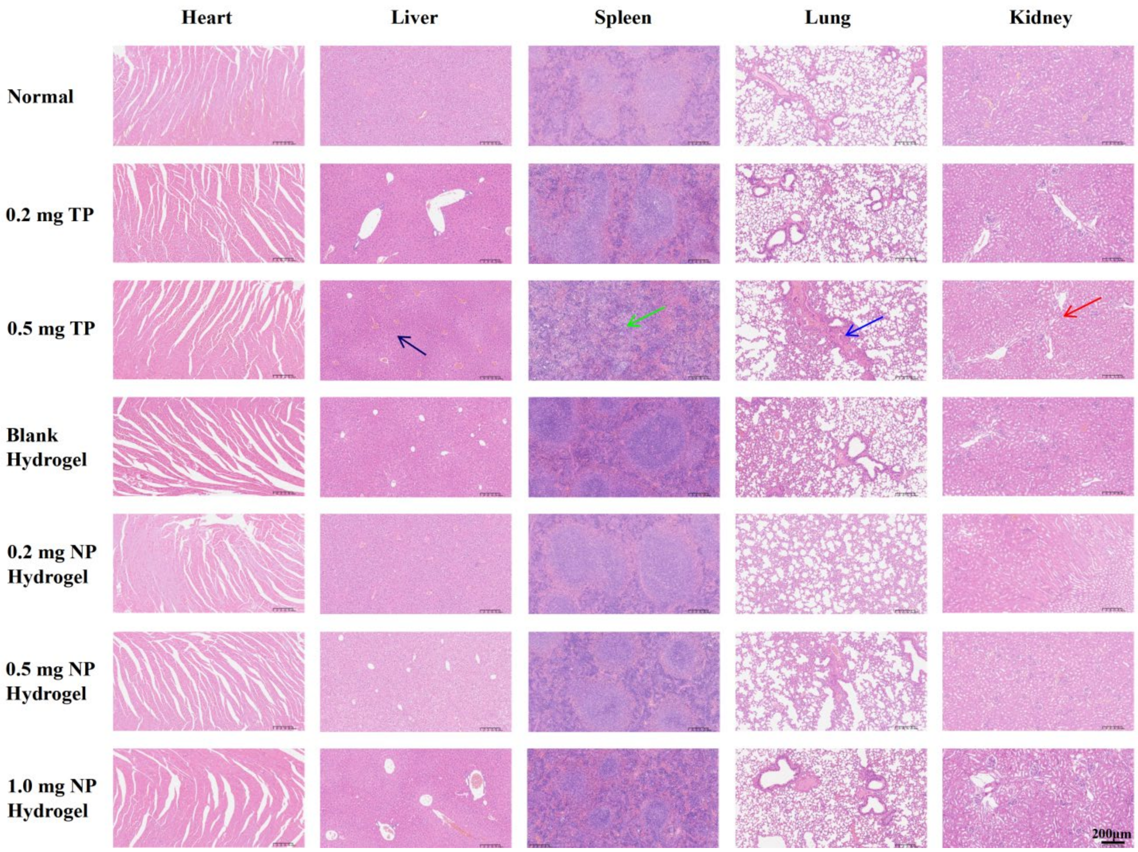

Figure S2. Hematoxylin and eosin (H&E) staining for biotoxicity of TP and TP@HSA NP hydrogels in each group. Black arrow: cell hypertrophy. Green arrow: blurred lymph node margin. Blue arrow: invasion of inflammation. Red arrow: size increased renal cell tubule vacuoles. (4×; scale bar: 200 μm).
